# Laboratory testing and phylogenetic analysis during a mumps outbreak in Ontario, Canada

**DOI:** 10.1186/s12985-018-0996-5

**Published:** 2018-06-05

**Authors:** Arnaud G. L’Huillier, Alireza Eshaghi, C. Sarai Racey, Katherene Ogbulafor, Ernesto Lombos, Rachel R. Higgins, David C. Alexander, Erik Kristjanson, Jocelyn Maregmen, Jonathan B. Gubbay, Tony Mazzulli

**Affiliations:** 1 Hospital for Sick Children, 555 University Avenue, Toronto, Ontario M5G 1X8 Canada; 2 Public Health Ontario, 661 University Avenue, Toronto, Ontario M5G 1M1 Canada; 3 Present address: Dalla Lana School of Public Health, 155 College Street, Toronto, Ontario M5T 3M7 Canada; 4 Present address: Cadham Provincial Laboratory, Winnipeg, Manitoba R3C 3Y1 Canada; 5 University of Toronto, 27 King’s College Circle, Toronto, Ontario M5S 1A1 Canada; 6 Mount Sinai Hospital, 600 University Avenue, Toronto, Ontario M5G 1X5 Canada

**Keywords:** Mumps, Outbreak, PCR, Serology, Viral culture, Diagnosis, Phylogeny

## Abstract

**Background:**

In September 2009, a mumps outbreak originated in New York and spread to Northeastern USA and Canada. This study compares the performance of different diagnostic testing methods used in Ontario and describes molecular characteristics of the outbreak strain.

**Methods:**

Between September 2009 and February 2010, specimens from suspect cases were submitted to Public Health Ontario Laboratory for mumps serology, culture and/or real-time reverse-transcriptase PCR (rRT-PCR) testing. rRT-PCR-positive specimens underwent genotyping at Canada’s National Microbiology Laboratory. Whole genome sequencing was performed on four outbreak and three sporadic viral culture isolates.

**Results:**

Six hundred ninety-eight patients had IgM serology testing, of which 255 (37%) had culture and rRT-PCR. Among those, 35/698 (5%) were IgM positive, 39/255 (15%) culture positive and 47/255 (18%) rRT-PCR-positive. Buccal swabs had the highest rRT-PCR positivity (21%). The outbreak isolates were identical to that in the New York outbreak occurring at the same time. Nucleotide and amino acid identity with the Jeryl Lynn vaccine strain ranged from 85.0-94.5% and 82.4-99.4%, depending on the gene and coding sequences. Homology of the HN protein, the main immunogenic mumps virus protein, was found to be 94.5 and 95.3%, when compared to Jeryl Lynn vaccine major and minor components, respectively.

**Conclusions:**

Despite higher sensitivity than serology, rRT-PCR testing is underutilized. Further work is needed to better understand the suboptimal match of the HN gene between the outbreak strain and the Jeryl Lynn vaccine strain.

## Background

Mumps virus is a single-stranded negative sense RNA virus, and a member of the *Paramyxoviridae* family. This usually self-limited disease is endemic worldwide and occurs primarily in children and adolescents. Twenty-five to 30% of infections are asymptomatic [1]. The most frequent clinical presentation is nonsuppurative parotitis, which occurs in up to 60-70% of persons infected [1]. Meningitis, oophoritis and epididymo-orchitis represent the most common complications [2]. Symptoms and complications might vary between immunized and unimmunized patients. Diagnosis relies on serology, viral culture, or molecular testing, such as real-time reverse-transcription polymerase chain reaction (rRT-PCR). If available, rRT-PCR is recommended on all patients being tested during the acute phase of illness because it has greater sensitivity than serology and, to a lesser extent, culture, as well as a faster turnaround time than culture [3–6]; moreover, serology has a low positive predictive value in a low prevalence setting [7]. Although immunization with the live-attenuated mumps vaccine (Jeryl Lynn strain), first licenced in the United States in 1967, and in Canada in 1969, has markedly reduced the incidence of mumps to around 0.1 cases per 100,000 persons/year, outbreaks still occur [8]. During the summer of 2009, a mumps outbreak that originated in New York State subsequently spread to Northeastern USA and Ontario, Canada [2, 9]. One hundred and thirty four cases of mumps were identified in Ontario, which has a population of 13 million inhabitants. The outbreak continued until June 2010 [9]. As the microbiology reference laboratory for Ontario, Public Health Ontario Laboratory (PHOL) provides all mumps diagnostic testing for the province, including testing of all clinical specimens related to this outbreak. The aim of this study was to compare the performance of different diagnostic tests among patients under investigation for mumps in a population with high immunization rates, review testing practices of clinicians in Ontario, and describe the molecular characteristics of the mumps virus strain causing Ontario’s 2009/2010 outbreak.

## Methods

All clinical specimens from suspected mumps cases in the province of Ontario were sent to PHOL for testing, which included serology, and both viral culture and mumps rRT-PCR on buccal, oral/oropharyngeal, nasopharyngeal, throat/pharyngeal, urine, sputum and cerebrospinal fluid (CSF) specimens. Persons under investigation were classified using the Ontario Ministry of Health provincial case definition for mumps [10], which was subsequently updated in 2014 [11]. Clinical specimens of patients with suspected mumps submitted to PHOL for testing between September 1, 2009 and February 22, 2010 were included in the analysis. Patients with confirmed mumps based on clinical data and epidemiological link without laboratory testing were therefore not included in the study. Blood specimens submitted for diagnostic serology were tested for mumps IgG and IgM by a commercial indirect enzyme-linked immunoassay (ELISA) kit (Enzygnost Anti-Parotitis ELISA, Siemens AG, Munich, Germany). Qualitative IgG seroconversion was assessed if more than one serial blood specimen was submitted (an IgG-negative specimen followed by an IgG positive specimen collected at a later date). Respiratory, urine, and CSF specimens were tested by viral culture and rRT-PCR. Specimens submitted for virus culture were inoculated in rhesus monkey kidney primary cell lines and either WI-38 or MRC5 human embryonic lung fibroblast diploid cell lines (Quidel Corporation, San Diego, CA). Identification of mumps virus was performed by monitoring for cytopathic effect and haemadsorption using 0.6% guinea pig red blood cells. Cell lines showing syncytia or a positive haemadsorption test were tested by an indirect immunofluorescent assay (IFA) for confirmation; more specifically, the IFA used a mumps monoclonal murine IgG1k antibody made against the Enders mumps strain and targeting two different epitopes of the fusion protein (Light Diagnostics™ Mumps IFA Kit, EMD Millipore Corporation, CA, USA). Initially negative cultures were passaged after 10 days, and reported negative following a further 7 days incubation (total incubation time 17 days). Cultured specimens were only considered mumps-positive if the mumps antigen was detected by IFA. The rRT-PCR was conducted at PHOL using an in-house assay adapted from protocols developed by Canada’s National Microbiology laboratory (NML) and the US Centers for Disease Control (CDC), which targets the fusion (F1) and small hydrophobic (SH) genes [12, 13]. Genotyping was performed at NML using RT-PCR and sequencing of the entire small hydrophobic gene (316 nucleotides) [14]. Whole genome sequencing (WGS) was performed on four outbreak and three sporadic viral culture Ontario isolates using overlapping primers folllowed by either next-generation sequencing using the Illumina MiSeq (Illumina Inc. San Diego, California, USA) or Sanger sequencing using the ABI 3730 DNA Analyzer (Applied Biosystems ™, Foster City, California, USA), as previously described [15] and compared to mumps reference strains [16].

Comparisons of assay’s sensitivity were performed using McNemar’s or Chi-square tests using SAS statistical software (version 9.2). *P*-values < 0.05 were considered significant.

## Results

### Diagnostic testing

Eight hundred and fifty-three blood specimens were received from 698 patients for IgM serology testing, of which 255 patients had 372 specimens submitted for both viral culture and rRT-PCR. Among those, 35/698 tested patients (5%) were IgM-positive, 39/255 tested patients (15%) were culture positive for mumps virus, and 47/255 tested patients (18%) were rRT-PCR-positive (Table 1). The most frequent specimen collection site for rRT-PCR was buccal (*n* = 158; 42.5% of rRT-PCR specimens), followed by urine (*n* = 146; 39.3% of rRT-PCR specimens). IgM serology was positive in 5.7% (49/853) of tested specimens whereas culture and rRT-PCR were positive in 13% (47/372) and 17% (62/372) tested specimens, respectively. Buccal swab rRT-PCR had the highest positivity (21%) among all specimen types (Table 1). Twenty-six percent (23/90) of patients who had more than one serum specimen submitted demonstrated mumps IgG seroconversion.

**Table 1:** Summary of specimens tested for mumps by serology, viral culture and rRT-PCR

	Specimens positive/tested (% pos)^b^	Persons positive/tested (% pos)^c^
IgM Serology	49/853 (6%)	35/698 (5%)
Viral Culture	47/372 (13%)	39/255 (15%)
rRT-PCR	62/372 (17%)	47/255 (18%)
Buccal	33/158 (21%)	
Urine	19/146 (14%)	
Oral/Oropharyngeal	1/11 (9%)	
Throat/Pharyngeal	3/24 (13%)	
Nasopharyngeal	0/8 (0%)	
CSF	1/1 (100%)	
Sputum	0/1 (0%)	
Other swab^a^	5/23 (22%)	

*rRT-PCR* real-time reverse-transcription polymerase chain reaction, *CSF* cerebrospinal fluid

^a^Collection site not provided

^b^Serology vs culture: *P* < 0.0001; Serology vs rRT-PCR: *P* < 0.0001; Culture vs rRT-PCR: *P* = 0.12

^c^Serology vs culture: *P* < 0.0001; Serology vs rRT-PCR: *P* < 0.0001; Culture vs rRT-PCR: *P* = 0.344

Assay comparison was conducted for all patients who had more than one test done and showed that rRT-PCR was superior to serology (Table 2); it also confirmed that buccal rRT-PCR was superior to urine rRT-PCR.

**Table 2:** Comparison of test performance in mumps positive patients tested by more than one testing modality

Positive mumps test (denominator)	Comparator test positivity (numerator)					
	Buccal rRT-PCR	Urine rRT-PCR	Any rRT-PCR	Any virus culture	IgM	IgG seroconversion
Buccal rRT-PCR positive		14/27 (51.9%)	33/33 (100%)	29/33 (87.9%)	8/24 (33.3%)	4/23 (17.4%)
Urine rRT-PCR positive	14/17 (82.4%)		19/19 (100%)	15/18 (83.3%)	8/14 (57.1%)	3/25 (12.0%)
Any rRT-PCR positive	33/40 (82.5%)	19/36 (52.8%)		37/45 (82.2%)	13/31 (41.9%)	6/30 (20.0%)
Any virus culture positive	29/33 (87.9%)	15/29 (51.7%)	37/39 (94.9%)		9/27 (33.3%)	4/30 (13.3%)
IgM positive	8/16 (50.0%)	8/14 (57.1%)	13/19 (68.4%)	9/17 (52.9%)		10/90 (11.1%)
IgG seroconversion	4/8 (50.0%)	3/10 (30.0%)	6/10 (60%)	4/9 (44.4%)	10/23 (43.5%)	

*rRT-PCR* real-time reverse-transcription polymerase chain reaction

Among the 47/255 rRT-PCR positive patients, 31 (66%) had an IgM test performed, of which 13 (42%) were positive, 14 (45%) negative, and four (13%) indeterminate. Surprisingly, only 19/35 (54%) IgM positive patients had specimens submitted for rRT-PCR and/or culture.

Among the 105 patients who had both buccal and urine specimens submitted, rRT-PCR was positive in buccal and/or urine specimen in 30 (29%). rRT-PCR was positive for buccal specimens only, urine specimens only and both urine and buccal specimens in 13, 3 and 14 cases, respectively; therefore, buccal swabs detected 27/30 (90%) of rRT-PCR positive specimens, compared to 17/30 (57%) for urine (*p* = 0.02). Time between symptom onset and collection date was poorly reported. Only 12.4% (64/517) of patients who were IgM negative, and had no rRT-PCR or culture testing done, had time since symptom onset reported. Median duration from illness onset to collection date in this group was 4 days (range 0-27 days). For IgM-positive patients, 13/35 (37%) had a reported time since symptom onset, with a median duration from illness onset to collection date of 4 days (range 3-26 days).

### Phylogenetic analysis

**Fig. 1:**
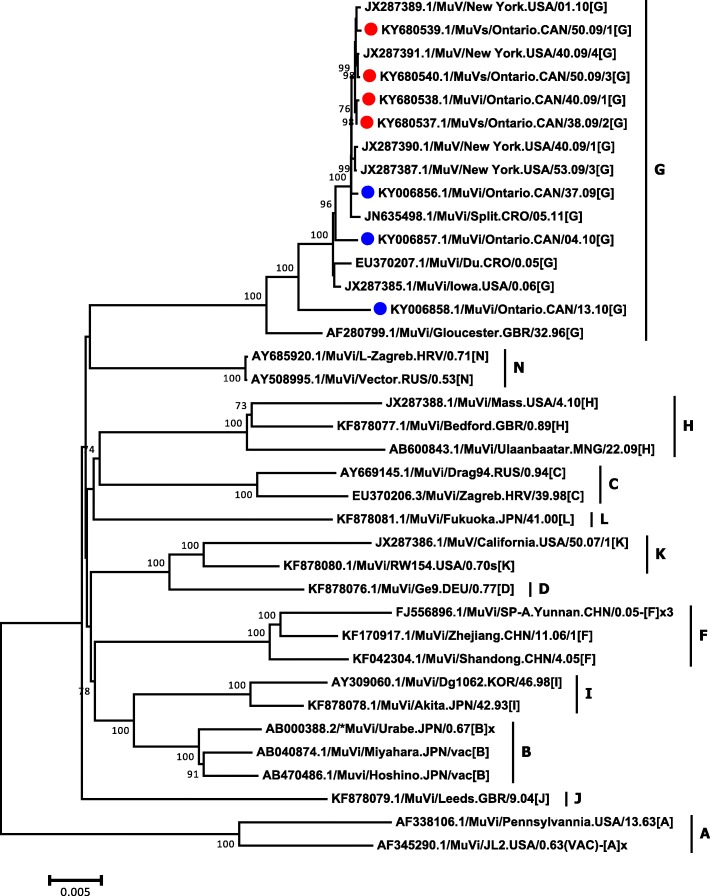
Whole genome sequencing (WGS) phylogenetic tree. The WGS phylogenetic tree was constructed using the nearly complete genomes (15,264 nucleotides) of four genotype G mumps outbreak isolates from Ontario (GenBank accession numbers: KY680537.1, KY680538.1, KY680539.1 and KY680540.1), three sporadic Ontario isolates (GenBank accession numbers: KY006856.1, KY006857.1 and KY006858.1) and representatives of other genotypes available in NCBI’s GenBank sequence database. The phylogenetic tree containing 37 strains was reconstructed using the Neighbor-Joining method and Maximum Composite Likelihood was used to compute the evolutionary distances. The statistical significance of the phylogenies constructed was estimated by bootstrap analysis of 1000 pseudo-replicate data sets (bootstrap values of > 70% are given at each node). The horizontal length of the bar denotes the nucleotide changes per site (MEGA version 6.06 software package). Outbreak and non-outbreaks isolates identified at Public Health Ontario Laboratory in 2010 are labelled with red and blue circles respectively

**Fig. 2:**
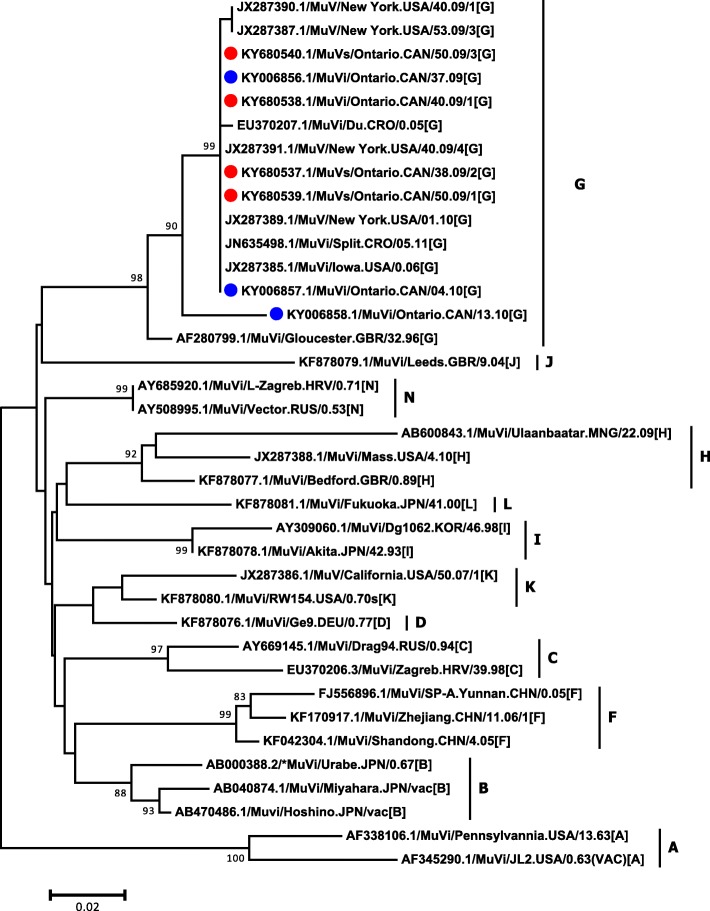
Small hydrophobic (SH) gene sequencing phylogenetic tree. The SH phylogenetic tree was constructed based on the 316 nucleotides of the entire SH gene of four genotype G mumps outbreak isolates from Ontario (GenBank accession numbers: KY680537.1, KY680538.1, KY680539.1 and KY680540.1), three sporadic Ontario isolates (GenBank accession numbers: KY006856.1, KY006857.1 and KY006858.1) and representatives of other genotypes available in NCBI’s GenBank sequence database. The phylogenetic tree containing 37 strains was reconstructed using the Neighbor-Joining method and Maximum Composite Likelihood was used to compute the evolutionary distances. The statistical significance of the phylogenies constructed was estimated by bootstrap analysis of 1000 pseudo-replicate data sets (bootstrap values of > 70% are given at each node). The horizontal length of the bar denotes the nucleotide changes per site (MEGA version 6.06 software package). Outbreak and non-outbreaks isolates identified at Public Health Ontario Laboratory in 2010 are labelled with red and blue circles respectively

Genotyping of the SH gene, performed at the Public Health Agency of Canada’s NML*,* confirmed that all tested outbreak and sporadic strains were genotype G; all were > 99% identical to a predominant circulating strain identified in Canada in 2007, with a difference of not more than two nucleotides. The four mumps outbreak isolates which underwent WGS (GenBank accession numbers: KY680537.1, KY680538.1, KY680539.1 and KY680540.1) had between 99.8-99.9% nucleotide identity with sequences from the New York 2009-2010 outbreak (GenBank accession numbers: JX287389.1, JX287391.1, JX287390.1 and JX287387.1), confirming that the same strain was circulating for the Ontario, Canada and New York outbreaks (Figs. 1 and 2, Table 3). Fifteen synonymous single-nucleotide polymorphisms were observed within the whole genome of Ontario’s outbreak strains and nucleotide identity of 99.9% was obtained. However, nucleotide identity within all seven Ontario strains (including non outbreak strains KY006856.1, KY006857.1 and KY006858.1) were calculated to be 98.7-99.8%. Nucleotide and amino acid identity between the Ontario outbreak strains and the Jeryl Lynn vaccine strain components varied between genes, ranging from 85.0 to 94.5% and from 82.4 to 99.4%, respectively (Table 4). Homology of the HN protein, the main immunogenic mumps virus protein, was found to be 94.5 and 95.3%, when compared to Jeryl Lynn vaccine major and minor components, respectively.

**Table 4:** Identity between Jeryl Lynn vaccine mumps strain and Ontario mumps whole genome sequences

Gene	JL Major % identity		JL Minor % identity	
	Nucleotide (%)	Amino acid (%)	Nucleotide (%)	Amino acid (%)
Nucleocapsid Protein (NP)	94.4	98.3	94.5	98.3
Phosphoprotein (P)	92.7–92.8	89.6–89.9	91.9–92.0	87.5–87.8
Matrix (M)	93.2–93.3	99.2	92.9–93.1	99.4
Fusion (F)	92.9–93.1	94.7–95.1	94.3–94.5	96.0–96.4
Small Hydrophobic (SH)	87.3	85.9	85.0	82.4
Hemagglutinin–Neuraminidase (HN)	91.0	94.5	91.9	95.3
Large Protein (L)	94.0	98.8–98.9	94.2–94.3	98.7–98.8

*JL* Jeryl Lynn

**Table 3:**
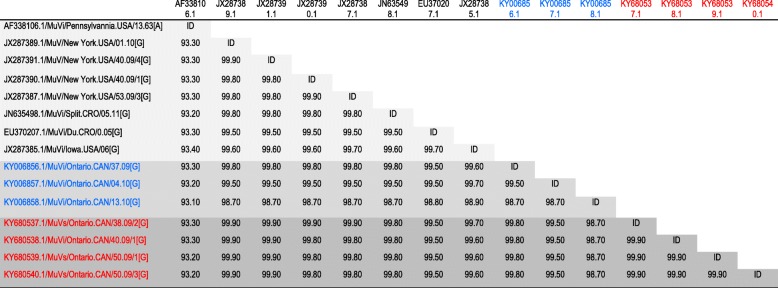
Percentage of whole genome sequence identity between mumps sequences

Outbreak and non-outbreaks isolates identified at Public Health Ontario Laboratory in 2010 are labelled in red and blue respectively

## Discussion

Mumps outbreaks continue to occur worldwide, including in populations with adequate immunization schedules and coverage. In 2016, more than 6000 cases of mumps were reported in the USA, the highest in 10 years [17]. In 2017, more than 5600 cases were reported in the USA, mostly related to an outbreak also involving Canada [17]. The outbreak that started in New York in 2009 and subsequently reached Ontario is one of the biggest reported outbreaks since universal immunization, with more than 3500 reported cases in the USA alone [2]. Phylogenetic analyses confirmed that the strain responsible for the outbreak in Ontario was similar to the one causing the outbreak in New York [2]. Moreover, WGS confirmed that outbreak isolates were > 98.7% similar to sporadic isolates, suggesting that the outbreak strain has been previously circulating. The presumed index case of the New York outbreak was a child who recently returned from the United Kingdom where a large outbreak was happening [18].

The outbreak in Ontario occurred in a population where 14% of cases were unimmunized, whereas 55 and 28% of cases had previously received 1 and 2 doses, respectively [9]. Therefore, the outbreak occured in a context of suboptimal immunization as well as possible waning of immunity. The relatively low percentage of sequence homology between our strain and the Jeryl Lynn vaccine strain is related to the fact that they belong to genotypes G and A, respectively, which has been suggested as a potential cause of suboptimal vaccine effectiveness [19, 20]. However, additional studies have shown that vaccine-induced antibody effectively neutralizes wild-type virus [19, 21, 22]. Consequently, the pathophysiology of mumps vaccine failure is still unclear.

Our study demonstrated that rRT-PCR was the most sensitive assay for mumps detection. Viral culture was slightly less sensitive, and this difference did not reach statistical significance. However, IgM serology was significantly less sensitive than both rRT-PCR and viral culture. Our observed order of assay sensitivity is consistent with large studies with > 80 laboratory confirmed cases [3, 5, 23, 24]. In addition, a number of studies have shown a better sensitivity of rRT-PCR compared to IgM serology [4, 25–32] and, to a lesser extent, culture [12, 25, 26]. However, inconsistent findings in smaller studies has been observed where IgM yielded similar or better sensitivity than culture [25, 26, 33] or RT-PCR [34]. More than 3500 cases were confirmed in the USA during the 2009-2010 outbreak that reached Ontario [2]. Among the 1648 tested patients in the USA, 831 (50%) were positive by at least one assay. In this large sample, rRT-PCR was the most sensitive assay with 68% positivity (373/550) among cases who had rRT-PCR testing, compared to 64% (283/443) and 35% (550/1563) of cases who underwent cell culture and IgM testing, respectively. The discrepant sensitivity of RT-PCR, culture and serology between the above-mentioned studies could be a result of confounding by the different vaccination status and different time between symptom onset and specimen collection among the studied populations. Indeed, previously immunized patients are less likely to develop an IgM response when subsequently exposed to mumps [2, 6]. Moreover, rRT-PCR and culture are more likely to be positive if specimens were collected in the first 2 days after parotitis onset [2, 6], whereas IgM is more likely to be positive if collected ≥ 3 days after symptom onset [6]. Among the same population, Rota et al. showed that sensitivity of rRT-PCR was higher than serology from day 0 to day 2 after symptom onset, whereas the sensitivity of serology was higher than rRT-PCR from day 3 onwards [6]. The fact that rRT-PCR yield was highest during day one to three after parotitis onset and decreased afterwards was confirmed in two other studies, using rRT-PCR [27] and RT-PCR [23], respectively.

Additional testing showed that rRT-PCR and IgM sensitivity varied significantly using different assays [6, 35]. In the Ontario outbreak, negative IgM tests in our confirmed cases could not be explained by early collection of specimens, as we observed a median duration of 4 days from symptom onset to blood collection, which should have been ample time for IgM to develop. The low sensitivity of IgM serology may also be confounded by immunization status. It has been suggested that immunized individuals have modified B cell responses that allow for the rapid generation of IgG antibodies and a blunted, or absent IgM response, when exposed to mumps virus [24]. This phenomenon could also result in a lack of IgG seroconversion being observed following mumps infection in previously immunized patients, due to an IgG boost occurring prior to clinical presentation. Studies performed in the USA during the 2009-2010 outbreak showed that IgM serology was less likely to be positive among immunized (26%) than unimmunized patients (76%, *P* < 0.05), whereas immunization status had no influence on rRT-PCR and culture results [2, 6]. This low yield of IgM in immunized patients during an outbreak has been reported in other studies [24, 36].

Our study also confirmed that rRT-PCR sensitivity varied with specimen type and was higher for buccal than oropharyngeal swabs or urine. This is consistent with previous studies showing that saliva swabs performed better than pharyngeal swabs, whereas test sensitivity was lowest for urine [4, 23, 37]. Similarly, Hindiyeh et al. showed that RT-PCR sensitivity was higher with pharyngeal or parotid fluid than urine [38].

The main limitation of the study is the relative lack of clinical data about the studied population, such as symptoms at disease onset. This is related to the fact that clinicians in the community forward specimens to our laboratory for mumps testing, often without providing significant clinical data. As a consequence, it was not possible to know precisely the immunization status of the tested patients which would have allowed comparison of diagnostic methods between immunized and unimmunized patients; however, the immunization status of the same population at this time was reported in another publication, showing that 86% of the population had received at least one dose and more than 55% two doses [9]. Similarly, the time between symptom onset and specimen collection was provided for a minority of patients only. As these data might influence serology and RT-PCR sensitivity, it would have provided important additional information in this setting. As it has been shown that a rRT-PCR targeting the nucleoprotein gene was more sensitive than a rRT-PCR targeting the SH gene [6], the fact that our assay targeted the F1 and SH genes might also be a potential limitation of the study.

Despite the abundance of previous data showing that RT-PCR, and to a lesser extent viral culture, are the most sensitive detection methods, most patients in our study had serology testing during the acute illness without any rRT-PCR or culture testing. This testing pattern was also observed in the US outbreak and may reflect the current practice in North America. During the same outbreak in the USA, IgM serology was used in 95% (*n* = 1563) of the 1648 tested patients, whereas rRT-PCR and viral culture were used in only 33% (*n* = 550) and 27% (*n* = 443), respectively [2]. This testing practice is in contrast to a 2009 Australian study that showed that RT-PCR was the most frequently used diagnostic method during a mumps outbreak in that country [3]. The current CDC guidelines recommend to perform serology as well as rRT-PCR or culture, but emphasize the lower yield of urine compared to buccal specimens [39]. On the other hand, the Public Health Agency of Canada recommends to perform serology as well as buccal and urine rRT-PCR but no longer recommends culture in the current guidelines [40]. Clinician education is needed to increase awareness of the availability, better sensitivity and test performance of rRT-PCR compared to serology.

Compared to rRT-PCR, IgM serology has lower sensitivity and may be highly influenced by immunization status. As such, rRT-PCR should be considered as the gold standard for mumps detection because of its high sensitivity and its quick turnaround time. However, it is not uncommon that patients with negative rRT-PCR [27, 29, 32] or RT-PCR [41], have positive IgM. As a consequence, these two assays are somewhat complementary, as IgM yield increases with time following symptom onset, when RT-PCR yield decreases. However, clinicians should keep in mind the poor positive predictive value of IgM when used in a low prevalence setting such as North America, for example in the absence of an outbreak [7]. Due to the impact of time since symptom onset and the relative sensitivity of rRT-PCR and serology, we therefore emphasize the need to continue educating healthcare providers of the importance of submitting both buccal swab and urine specimens for rRT-PCR, in addition to a specimen for IgM/IgG serology when mumps is suspected, in accordance with current guidelines.

## Conclusions

This study confirms that rRT-PCR has higher sensitivity than serology, and is still underutilized for the diagnosis of mumps. This work also confirms the superiority of buccal rRT-PCR compared to urine rRT-PCR. Phylogenetic analysis confirmed the discrepancies in nucleotide and amino acid homology between outbreak and vaccine strains. Further work is needed to better understand the suboptimal match of the HN gene between the outbreak strain and the Jeryl Lynn vaccine strain, in order to better protect vaccinated individuals.

## Abbreviations

CPE: Cytopathic effect
CSF: Cerebrospinal fluid
F1: Fusion gene
PHOL: Public Health Ontario Laboratory
rRT-PCR: Real-time reverse-transcriptase PCR
SH: Small hydrophobic gene
WGS: Whole genome sequencing

## References

[1] Hviid A, Rubin S, Muhlemann K (2008). Mumps. Lancet.

[2] Barskey AE, Schulte C, Rosen JB, Handschur EF, Rausch-Phung E, Doll MK (2012). Mumps outbreak in orthodox Jewish communities in the United States. N Engl J Med.

[3] Bangor-Jones RD, Dowse GK, Giele CM, van Buynder PG, Hodge MM, Whitty MM (2009). A prolonged mumps outbreak among highly vaccinated aboriginal people in the Kimberley region of Western Australia. Med J Aust.

[4] Maillet M, Bouvat E, Robert N, Baccard-Longere M, Morel-Baccard C, Morand P (2015). Mumps outbreak and laboratory diagnosis. J Clin Virol.

[5] Nelson GE, Aguon A, Valencia E, Oliva R, Guerrero ML, Reyes R (2013). Epidemiology of a mumps outbreak in a highly vaccinated island population and use of a third dose of measles-mumps-rubella vaccine for outbreak control--Guam 2009 to 2010. Pediatr Infect Dis J.

[6] Rota JS, Rosen JB, Doll MK, McNall RJ, McGrew M, Williams N (2013). Comparison of the sensitivity of laboratory diagnostic methods from a well-characterized outbreak of mumps in New York city in 2009. Clin Vaccine Immunol.

[7] Tuokko H (1984). Comparison of nonspecific reactivity in indirect and reverse immunoassays for measles and mumps immunoglobulin M antibodies. J Clin Microbiol.

[8] Dayan GH, Quinlisk MP, Parker AA, Barskey AE, Harris ML, Schwartz JM (2008). Recent resurgence of mumps in the United States. N Engl J Med.

[9] Deeks SL, Lim GH, Simpson MA, Gagne L, Gubbay J, Kristjanson E (2011). An assessment of mumps vaccine effectiveness by dose during an outbreak in Canada. CMAJ.

[10] The Ontario Ministry of Health and Long-Term Care. Mumps. Can Commun Dis Rep. 2009;35(S2):73–4.

[11] The Ontario Ministry of Health and Long-Term Care. Infectious Diseases Protocol. Mumps. Appendix B: Provincial Cases Definitions for Reportable Diseases. Revised January 2014. 2014. Available from: http://www.health.gov.on.ca/en/pro/programs/publichealth/oph_standards/docs/mumps_cd.pdf. Accessed 23 Jan 2018.

[12] Uchida K, Shinohara M, Shimada S, Segawa Y, Doi R, Gotoh A (2005). Rapid and sensitive detection of mumps virus RNA directly from clinical samples by real-time PCR. J Med Virol.

[13] Boddicker JD, Rota PA, Kreman T, Wangeman A, Lowe L, Hummel KB (2007). Real-time reverse transcription-PCR assay for detection of mumps virus RNA in clinical specimens. J Clin Microbiol.

[14] Mumps virus nomenclature update: 2012 (2012). Wkly Epidemiol Rec.

[15] Ma SLJ, Shi H, Wang L, Wang J, Liu L, Li Q (2009). Complete nucleotide sequence of a mumps virus SP strain isolated in China. Virol Sin.

[16] Jin L, Orvell C, Myers R, Rota PA, Nakayama T, Forcic D (2015). Genomic diversity of mumps virus and global distribution of the 12 genotypes. Rev Med Virol.

[17] Centers for Diseases Control and Prevention (CDC). Mumps cases and outbreaks. 2017. Available from:https://www.cdc.gov/mumps/outbreaks.html. Accessed 23 Jan 2018.

[18] Yung C, Bukasa A, Brown K, Pebody R. Public health advice based on routine mumps surveillance in England and Wales. Euro Surveill. 2010;15(38):19669. 10.2807/ese.15.38.19669-en 20929652

[19] Rubin SA, Qi L, Audet SA, Sullivan B, Carbone KM, Bellini WJ (2008). Antibody induced by immunization with the Jeryl Lynn mumps vaccine strain effectively neutralizes a heterologous wild-type mumps virus associated with a large outbreak. J Infect Dis.

[20] Nojd J, Tecle T, Samuelsson A, Orvell C (2001). Mumps virus neutralizing antibodies do not protect against reinfection with a heterologous mumps virus genotype. Vaccine.

[21] Santak M, Lang-Balija M, Ivancic-Jelecki J, Kosutic-Gulija T, Ljubin-Sternak S, Forcic D (2013). Antigenic differences between vaccine and circulating wild-type mumps viruses decreases neutralization capacity of vaccine-induced antibodies. Epidemiol Infect.

[22] Gouma S, Sane J, Gijselaar D, Cremer J, Hahne S, Koopmans M (2014). Two major mumps genotype G variants dominated recent mumps outbreaks in the Netherlands (2009-2012). J Gen Virol.

[23] Tan KE, Anderson M, Krajden M, Petric M, Mak A, Naus M (2011). Mumps virus detection during an outbreak in a highly unvaccinated population in British Columbia. Can J Public Health.

[24] Hatchette T, Davidson R, Clay S, Pettipas J, Leblanc J, Sarwal S (2009). Laboratory diagnosis of mumps in a partially immunized population: the Nova Scotia experience. Can J Infect Dis Med Microbiol.

[25] Whelan J, van Binnendijk R, Greenland K, Fanoy E, Khargi M, Yap K, et al. Ongoing mumps outbreak in a student population with high vaccination coverage, Netherlands, 2010. Euro Surveill. 2010;15(17):19554. 10.2807/ese.15.17.19554-en 20460086

[26] Cusi MG, Bianchi S, Valassina M, Santini L, Arnetoli M, Valensin PE (1996). Rapid detection and typing of circulating mumps virus by reverse transcription/polymerase chain reaction. Res Virol.

[27] Bitsko RH, Cortese MM, Dayan GH, Rota PA, Lowe L, Iversen SC (2008). Detection of RNA of mumps virus during an outbreak in a population with a high level of measles, mumps, and rubella vaccine coverage. J Clin Microbiol.

[28] Sartorius B, Penttinen P, Nilsson J, Johansen K, Jonsson K, Arneborn M (2005). An outbreak of mumps in Sweden, February-April 2004. Euro Surveill..

[29] Rota JS, Turner JC, Yost-Daljev MK, Freeman M, Toney DM, Meisel E (2009). Investigation of a mumps outbreak among university students with two measles-mumps-rubella (MMR) vaccinations, Virginia, September-December 2006. J Med Virol.

[30] Brockhoff HJ, Mollema L, Sonder GJ, Postema CA, van Binnendijk RS, Kohl RH (2010). Mumps outbreak in a highly vaccinated student population, the Netherlands, 2004. Vaccine.

[31] Montes M, Cilla G, Artieda J, Vicente D, Basterretxea M (2002). Mumps outbreak in vaccinated children in Gipuzkoa (Basque Country), Spain. Epidemiol Infect.

[32] Nedeljkovic J, Kovacevic-Jovanovic V, Milosevic V, Seguljev Z, Petrovic V, Muller CP (2015). A mumps outbreak in Vojvodina, Serbia, in 2012 underlines the need for additional vaccination opportunities for young adults. PLoS One.

[33] Marin M, Quinlisk P, Shimabukuro T, Sawhney C, Brown C, Lebaron CW (2008). Mumps vaccination coverage and vaccine effectiveness in a large outbreak among college students--Iowa, 2006. Vaccine.

[34] Raut CG, Sinha DP, Jayaprakash H, Hanumiah H, Manjunatha MJ (2015). Mumps disease outbreak in Davangere district of Karnataka, India. Indian J Med Microbiol.

[35] Krause CH, Eastick K, Ogilvie MM (2006). Real-time PCR for mumps diagnosis on clinical specimens--comparison with results of conventional methods of virus detection and nested PCR. J Clin Virol.

[36] Seaux L, Coucke L, Decruyenaere P, Padalko E (2015). Confusing mumps serology during an outbreak. J Clin Virol.

[37] Afzal MA, Buchanan J, Dias JA, Cordeiro M, Bentley ML, Shorrock CA (1997). RT-PCR based diagnosis and molecular characterisation of mumps viruses derived from clinical specimens collected during the 1996 mumps outbreak in Portugal. J Med Virol.

[38] Hindiyeh MY, Aboudy Y, Wohoush M, Shulman LM, Ram D, Levin T (2009). Characterization of large mumps outbreak among vaccinated Palestinian refugees. J Clin Microbiol.

[39] Parker Fiebelkorn A, Barskey A, Hickman C, Bellini WJ (2012). Manual for the surveillance of vaccine-preventable diseases. Chapter 9: mumps.

[40] Public Health Agency of Canada (PHAC) (2010). Guidelines for the prevention and control of mumps outbreaks in Canada. Can Commun Dis Rep.

[41] Reid F, Hassan J, Irwin F, Waters A, Hall W, Connell J (2008). Epidemiologic and diagnostic evaluation of a recent mumps outbreak using oral fluid samples. J Clin Virol.

